# Real-Time Robust 2.5D Stereo Multi-Object Tracking with Lightweight Stereo Matching Algorithm

**DOI:** 10.3390/s25216773

**Published:** 2025-11-05

**Authors:** Jinhyeong Lee, Junyoung Shin, Eunwoo Park, Daekeun Kim

**Affiliations:** 1 Department of Mechanical Engineering, Dankook University, Yongin 16890, Republic of Korea; jinhyeong0801@dankook.ac.kr (J.L.); jun4947@dankook.ac.kr (J.S.); eunwoopark@postech.ac.kr (E.P.); 2 Department of Convergence IT Engineering, Pohang University of Science and Technology (POSTECH), Pohang 37673, Republic of Korea

**Keywords:** stereo vision, multi-object tracking, stereo matching, depth estimation, stereo tracking, tracker re-identification, occlusion & truncation handling

## Abstract

**Highlights:**

**What are the main findings?**

- Lightweight stereo matching using only bounding box coordinates achieves robust multi-object tracking with a MOTA of 0.932 and an IDF1 of 0.823, outperforming state-of-the-art monocular trackers.
- A dual-tracker design with a re-identification mechanism maintains consistent object identities during occlusions and truncations by leveraging stereo redundancy.

**What are the implications of the main findings?**

- Resource-efficient 2.5D tracking enables real-time deployment (70 FPS) on standard hardware without expensive 3D reconstruction or dense stereo matching.
- Stereo vision’s inherent redundancy provides a practical solution for robust tracking in challenging real-world scenarios like retail monitoring and autonomous systems.

**Abstract:**

Multi-object tracking faces persistent challenges from occlusions and truncations in monocular vision systems. While stereo vision provides depth information, existing approaches require computationally expensive dense matching or 3D reconstruction. This paper presents a real-time 2.5D stereo multi-object tracking framework combining lightweight stereo matching with resilient tracker management. The stereo matching module employs Direct Linear Transform-based triangulation using only bounding box coordinates, eliminating costly feature extraction while maintaining robust correspondence through geometric constraints. A dual-tracker architecture maintains independent trackers in both views, enabling re-identification when objects become occluded in one view but remain visible in the other. Experimental validation on a refrigerator monitoring dataset demonstrates that StereoSORT achieves a multiple object tracking accuracy (MOTA) of 0.932 and an identification F1 score (IDF1) of 0.823, substantially outperforming monocular trackers, including OC-SORT (IDF1: 0.765) and ByteTrack (IDF1: 0.609). The system achieves a 50.1 mm median depth error, comparable to commercial sensors, while maintaining 70 FPS on standard hardware. These results validate that geometric constraints alone enable robust stereo tracking without appearance features, offering a practical solution for resource-constrained environments where computational efficiency and tracking reliability are equally critical.

## 1. Introduction

Multi-object tracking (MOT) is a fundamental technique in tracking and perception that aims to estimate the states of multiple objects over time while preserving their unique identities (IDs) [1]. MOT has been extensively studied in the broader tracking community through classical MOT approaches, such as Multiple Hypothesis Tracking (MHT) [2] and Joint Probabilistic Data Association (JPDA) [3], which address data association under uncertainty. With the development of deep learning-based object detectors, MOT has evolved within computer vision under the tracking-by-detection paradigm [4], where objects are detected in each frame and temporally associated. Among them, three-dimensional (3D) MOT [5,6,7] has become increasingly important in diverse real-world domains—ranging from autonomous driving and robotic navigation to surveillance and augmented reality [8,9,10,11]. While monocular vision systems [12,13] have shown remarkable progress with the advancement of deep learning techniques, they inherently lack depth information, limiting their ability to provide accurate 3D localization [14]. Compared to LiDAR-based systems [15,16], which provide highly accurate depth measurements but require expensive hardware and significant power consumption, stereo vision systems offer a cost-effective alternative for acquiring depth information via triangulation, making it particularly attractive for real-time 3D tracking where cost and power efficiency are critical [17,18].

The integration of deep learning-based object detection with stereo vision has enabled true 3D tracking capabilities beyond the limitations of image-based 2D tracking. Recent state-of-the-art detectors [19] achieve high accuracy in detecting and classifying objects with bounding box localization, but stereo vision can transform these 2D detections into 3D spatial information through depth estimation. The primary challenge in stereo vision-based tracking lies in establishing reliable correspondences between detected objects across stereo image pairs. This stereo matching process is critical for accurate depth estimation and subsequent 3D localization through triangulation [20,21]. Beyond accurate depth recovery, stereo vision also offers an inherent advantage over monocular systems: the disparity between viewpoints can mitigate occlusions or truncations in one view by leveraging better visibility in the other view.

However, despite recent advances in deep learning-based object detection, these systems remain susceptible to misdetection, spurious detections, misclassification, occlusion, and truncation, particularly in complex real-world scenarios [22]. These detection errors can significantly degrade stereo matching accuracy, leading to unreliable depth estimation. When objects are partially occluded or truncated at image boundaries, traditional tracking systems struggle to maintain consistent object IDs, often leading to tracking failures and ID switches [23,24]. While stereo vision’s viewpoint redundancy offers theoretical advantages, conventional stereo matching algorithms fail to fully exploit this potential when at least one view experiences detection errors. Traditional approaches attempt to mitigate these issues through additional shape similarity measures or feature descriptors [25,26], but such methods significantly increase computational overhead, making them impractical for resource-constrained environments.

The gap between the theoretical advantages of stereo vision and its practical limitations motivates the need for a more efficient approach. Rather than pursuing computationally expensive full 3D reconstruction [5], we recognize that many practical applications primarily require accurate depth-aware localization [27] rather than complete geometric modeling [28]. This insight leads us to adopt a 2.5D representation that combines 2D bounding boxes with depth information, eliminating the computational burden of 3D bounding box regression while maintaining essential spatial awareness for real-world applications.

In this paper, we propose a real-time 2.5D multi-object tracking system that achieves robust performance against occlusions and truncations through a novel stereo matching algorithm. Our system consists of two core components: a lightweight stereo matching module and a robust stereo tracking module. The stereo matching module evaluates all possible object correspondences between stereo image pairs by computing depth information through triangulation based on bounding box coordinates. For each potential match, we apply disparity-based image warping to align the bounding boxes and calculate their Intersection over Union (IoU) scores. The Hungarian algorithm then determines the optimal multi-object correspondence configuration, ensuring globally consistent matching without requiring computationally expensive feature extraction. The stereo tracking module creates independent trackers for each matched object in both stereo views, operating in parallel to maintain tracking continuity. This dual-tracker approach ensures that object tracking persists as long as at least one tracker remains active, effectively handling scenarios where objects become occluded or truncated in one view but remain visible in the other.

The main contributions of this work are threefold. First, we introduce a resource-aware stereo matching algorithm that achieves high matching accuracy using only detected bounding box coordinates without computing shape-based similarity or extracting appearance features. This minimalistic approach dramatically reduces computational overhead while maintaining robust correspondence-matching performance through the synergistic use of geometric constraints and disparity-driven spatial alignment, proving that these fundamental stereo vision principles are sufficient for reliable object matching. Second, we develop a resilient stereo tracking management system that can be seamlessly integrated with most existing object tracking algorithms. Our dual-tracker design provides exceptional robustness: when one tracker is lost due to detection errors, the system continues tracking with the remaining tracker, and once the detection issue is resolved, the lost tracker can be automatically recovered and synchronized with the existing one. This recovery mechanism ensures tracking continuity even under frequent detection failures. Third, we demonstrate that our 2.5D representation, combining the simplicity of 2D bounding boxes with essential depth information, provides practical advantages over full 3D MOT systems. Extensive experiments validate that our approach achieves comparable or superior tracking performance to state-of-the-art methods while requiring significantly fewer computational resources, enabling real-time operation on standard hardware platforms.

The remainder of this paper is organized as follows. Section 2 reviews related work on stereo tracking, object detection, stereo matching, and visibility handling. Section 3 presents our proposed methodology, including the detailed design of the lightweight stereo matching module and the resilient stereo tracking module. Section 4 describes the implementation details (experimental setup and datasets) and presents comprehensive experimental results and analysis, including comparisons with state-of-the-art methods and computational efficiency evaluations. Section 5 discusses the implications of our findings and limitations. Finally, Section 6 concludes the paper and outlines future research directions.

## 2. Related Work

### 2.1. Stereo Vision-Based Object Tracking

Stereo vision-based object tracking, as defined by Geiger et al. [5] in the KITTI benchmark, employs synchronized stereo image pairs to continuously localize and identify objects over time. This approach exploits binocular disparity for depth extraction through triangulation between corresponding features, enabling 3D spatial understanding without active sensors.

Li et al. [29] proposed joint spatial–temporal optimization for stereo 3D object tracking, combining deep neural network detection with geometric bundle adjustment. Their method regresses initial 3D bounding boxes and predicts dense object cues, including local depth and coordinates. While achieving state-of-the-art performance on KITTI, the dense cue prediction and iterative optimization limit real-time capability.

Karaev et al. [30] introduced DynamicStereo for consistent dynamic depth estimation from stereo videos, focusing on temporal consistency through transformer architectures. Their framework handles dynamic scenes effectively but requires computationally expensive temporal aggregation networks. Additionally, the reliance on learned temporal patterns fails when objects undergo rapid motion changes or severe occlusions.

Zhang et al. [31] developed TemporalStereo, an efficient spatial–temporal stereo matching network for video sequences. Their coarse-to-fine approach leverages sparse cost volume and past geometry information to enhance matching accuracy. However, the method requires extensive training data and struggles with domain adaptation to new environments. The end-to-end learning paradigm also lacks interpretability when failures occur.

### 2.2. Deep Learning-Based Object Detection

Faster R-Convolutional Neural Network (CNN) by Ren et al. [32] established the two-stage detection paradigm with Region Proposal Networks (RPN) sharing convolutional features for end-to-end training. While achieving high accuracy is crucial for tracking systems, the two-stage pipeline limits real-time performance on resource-constrained platforms.

The YOLO series by Redmon et al. [33] revolutionized object detection by reformulating it as single-stage regression, eliminating the time-consuming region proposal step of two-stage detectors. This unified architecture processes entire images in a single forward pass, achieving real-time performance. YOLO has evolved rapidly through numerous versions, with recent implementations becoming the de facto standard for real-time detection applications. Despite its widespread adoption and continuous improvements, localization accuracy limitations and small object detection challenges persist, particularly in cluttered scenes.

### 2.3. Depth Estimation and Stereo Matching Algorithms

Scharstein and Szeliski [17] defined the four-stage stereo correspondence pipeline: matching cost computation, aggregation, disparity optimization, and refinement. They formalized triangulation-based depth recovery from disparity and established the Middlebury benchmark for algorithm evaluation.

Hirschmüller’s Semi-Global Matching (SGM) [18] remains the standard for dense disparity estimation, approximating global energy minimization through multiple one-dimensional path optimizations. While achieving accurate depth in textured regions with balanced computational efficiency, SGM fails in textureless areas and requires substantial resources for high-resolution processing.

Pyramid Stereo Matching Network (PSMNet) by Chang and Chen [34] employs spatial pyramid pooling and stacked hourglass architecture for multi-scale context aggregation. Despite state-of-the-art accuracy on benchmarks, the significant processing time per stereo pair prohibits real-time applications, and global context assumptions fail for dynamic scenes.

### 2.4. Multi-Object Tracking Under Occlusion and Truncation

The MOT16 benchmark by Milan et al. [35] established standardized evaluation protocols for occlusion and truncation handling in crowded scenes. The benchmark reveals occlusion as the primary performance bottleneck, with traditional motion and appearance-based methods failing in dense scenarios with similar objects.

DeepSORT by Wojke et al. [36] extends Simple Online and Realtime Tracking (SORT) with deep appearance features from person re-identification networks, combining Kalman filtering with learned visual representations for data association. While reducing ID switches in occlusion scenarios, the per-object feature extraction overhead limits scalability, and appearance features degrade severely at truncated image boundaries.

ByteTrack by Zhang et al. [24] recovers low-confidence detections typically discarded by other trackers, recognizing that occluded objects often produce weak detection responses. This strategy improves trajectory completeness but increases false positives in cluttered scenes and fails during extended complete occlusions due to temporal dependency.

Observation-Centric SORT (OC-SORT) by Cao et al. [37] introduces Observation-Centric Momentum (OCM) to maintain velocity consistency from historical observations rather than noisy motion predictions, achieving real-time performance and reducing ID switches. Despite improved robustness to detection noise, it remains vulnerable to extended occlusions and lacks appearance features for disambiguating similar objects.

Weng et al. [7] demonstrated that 3D MOT with A Baseline for 3D MOT (AB3DMOT) using 3D Kalman filtering and IoU association inherently handles occlusions through physical constraints. However, the dependency on accurate 3D detectors or expensive 3D bounding box regression limits practical deployment, as these detection requirements impose substantial computational overhead independent of the tracking algorithm itself.

Beyond traditional tracking-by-detection frameworks, Random Finite Set (RFS) methods [38] offer a principled probabilistic framework for MOT by handling both detection uncertainty and data association simultaneously. In particular, the Labeled RFS (LRFS) framework [39] extends RFS theory to maintain object identities over time and has demonstrated competitive performance through Gibbs sampling-based data association under occlusion and re-identification scenarios. Similar to our proposed method, LRFS also follows the tracking-by-detection paradigm by utilizing detection outputs as measurement inputs for state estimation. However, RFS-based approaches generally require iterative probabilistic inference, which increases computational complexity and limits real-time deployment. In contrast, this work focuses on achieving robust identity preservation in real-time by leveraging stereo geometric consistency without heavy probabilistic modeling.

## 3. Materials and Methods

### 3.1. Overview of the Proposed Framework

This study aims to develop a robust 2.5D multi-object tracking technology that is resilient to the object detection and tracking errors commonly encountered in real-world stereo vision applications. While achieving perfect accuracy in object detection and tracking remains practically impossible due to occlusions, illumination variations, motion blur, and appearance ambiguities, our framework addresses these challenges by leveraging the redundancy of stereo vision systems: when detection or tracking errors occur in one view, the complementary information from the other view can maintain tracking continuity. The framework employs geometric consistency verification through stereo triangulation and disparity-based validation to handle uncertainties in both detection and stereo matching while utilizing temporal information to predict object locations and recover from temporary failures [5,40].

**Figure 1:**
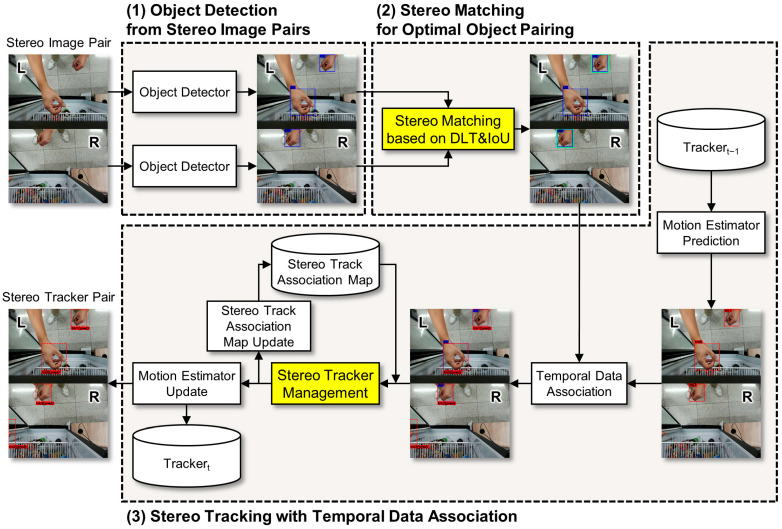
Overall architecture of the proposed 2.5D multi-object tracking framework. The system consists of three sequential stages: (1) Object Detection from Stereo Image Pairs, where independent detection is performed on synchronized left (L) and right (R) images; (2) Stereo Matching for Optimal Object Pairing, which establishes correspondences between detected objects using Direct Linear Transform (DLT)-based triangulation and IoU validation (highlighted in yellow); and (3) Stereo Tracking with Temporal Data Association, which maintains object IDs across frames through tracker management (highlighted in yellow), motion estimation, and stereo track association map updates. The feedback loop from tracking predictions enhances detection and matching in subsequent frames.

The proposed framework consists of three sequential stages: (1) object detection from stereo image pairs, using deep learning models to identify objects in both left and right images, (2) stereo matching for optimal object pairing across the stereo image pair, computing 2.5D positions through a combination of geometric triangulation and image disparity, and (3) stereo tracking with temporal data association, maintaining object IDs across frames and handling temporary occlusions through re-identification mechanisms. These stages are integrated through a feedback loop where tracking predictions enhance subsequent detection and matching processes [41]. Figure 1 illustrates the overall system architecture, showing the data flow through the three stages and the feedback mechanisms that enable robust tracking performance. Detailed descriptions of each stage are provided in the following subsections.

### 3.2. Stage 1: Object Detection from Stereo Image Pairs

The first stage performs object detection on synchronized stereo image pairs captured from a calibrated stereo camera system. Here, the term ‘stereo image pair’ refers only to the left and right images acquired simultaneously at each frame; object detection is applied independently to each image without using stereo relationships. As shown in Figure 1, an object detector is applied independently to each image, enabling parallel processing that preserves objects visible in only one view due to occlusions or limited field overlap. This independent detection strategy provides redundancy that enhances system robustness against single-view detection failures.

The detection model outputs, for each identified object, a class label, bounding box coordinates (x, y, width, and height), and a confidence score. These confidence scores are subsequently utilized in the stereo matching stage to weight matching decisions and resolve ambiguous correspondences. The state-of-the-art detection architecture ensures robust performance across varying environmental conditions. The object detection process is described in Algorithm 1 using pseudocode notation.
**Algorithm 1:** Stereo Object Detection (Pseudocode)
Input: Stereo image pair (IL, IR) at time t Output: Detection sets DL and DR 1: procedure STEREO_OBJECT_DETECTION(IL, IR) 2:   // Synchronize and preprocess images 3:   IL_sync, IR_sync ← SYNCHRONIZE(IL, IR) 4: 5:   // Initialize the detection sets 6:   DL ← ∅, DR ← ∅ 7: 8:   // Detection on left image 9:   DL ← DETECT_OBJECTS(IL_sync) 10:    for each detection dL in DL do 11:      dL.class ← CLASSIFY(dL) 12:      dL.bbox ← (xL, yL, wL, hL) 13:      dL.conf ← CONFIDENCE_SCORE(dL) 14:    end for 15: 16:    // Detection on right image 17:    DR ← DETECT_OBJECTS(IR_sync) 18:    for each detection dR in DR do 19:      dR.class ← CLASSIFY(dR) 20:      dR.bbox ← (xR, yR, wR, hR) 21:      dR.conf ← CONFIDENCE_SCORE(dR) 22:    end for 23: 24:    return DL, DR 25: end procedure

### 3.3. Stage 2: Stereo Matching for Optimal Object Pairing

**Figure 2:**
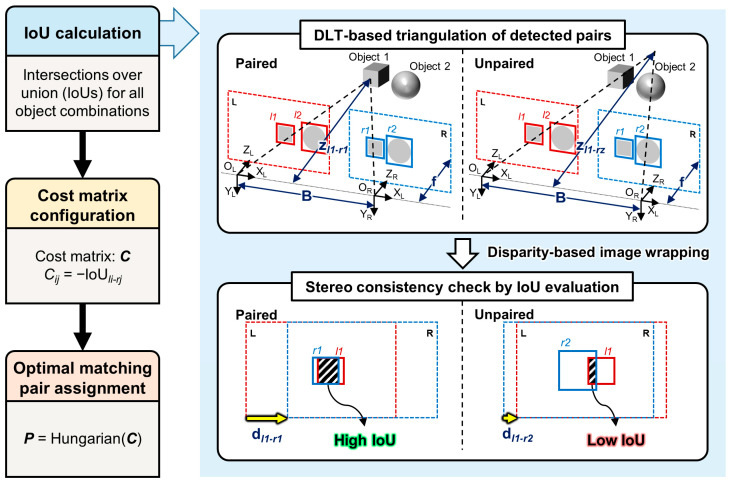
Stereo matching process using DLT-based triangulation and IoU validation. The method evaluates all possible object combinations between left (L) and right (R) detections. For each candidate pair, DLT-based triangulation estimates the 3D position assuming correspondence, from which the expected disparity is computed. Bounding boxes are then warped using the calculated disparity, and stereo consistency is verified through IoU evaluation. The cost matrix C with elements C*_ij_* = −IoU*_ij_* is constructed for all combinations, and the Hungarian algorithm determines optimal assignments. High IoU values indicate correct matches (paired), while low IoU values suggest incorrect correspondences (unpaired).

The second stage implements a stereo matching module for optimal object pairing through Direct Linear Transform (DLT)-based triangulation and stereo consistency verification, as illustrated in Figure 2. Unlike traditional appearance-based matching methods that rely on visual features, our approach leverages stereo geometry to validate object correspondences, making it more robust to appearance variations and illumination changes.

Given all possible object combinations between left and right detections (DL and DR from Stage 1), the algorithm performs DLT-based triangulation [20] for each candidate pair under the assumption that they represent the same physical object. The DLT method estimates the 3D position by solving an overdetermined system derived from the projection matrices of both cameras. From the triangulated 3D position (X, Y, Z), the expected disparity d (shown as $d_{l1-r1}$, $d_{l1-r2}$ in Figure 2) is computed using the fundamental stereo geometry relationship d=bf/Z, where b is the baseline and f is the focal length [17].

Using the computed disparity, each bounding box is warped to the opposite view through disparity-based image warping [42]. The stereo consistency is then evaluated by computing the IoU between the warped bounding box and the actual detection in the target image. This IoU metric quantifies the geometric consistency of the hypothesized correspondence [43]. Figure 2 demonstrates how high IoU values indicate correct matches (paired objects), while low IoU values suggest incorrect correspondences (unpaired objects).

A cost matrix C is constructed where each element $C_{ij}=-IoU_{ij}$ represents the negative IoU score between the i-th detection in the left image and the *j*-th detection in the right image. The negative sign transforms the maximization problem into a minimization problem suitable for the Hungarian algorithm [44]. The optimal assignment P = Hungarian(C) yields the one-to-one correspondence that maximizes the total IoU score. Pairs with high IoU values indicate correct matches, forming stereo object pairs—matched bounding boxes from left and right images corresponding to the same physical object. In contrast, low IoU values suggest incorrect correspondences. The stereo matching process is described in Algorithm 2 using pseudocode notation.
**Algorithm 2:** Stereo Object Matching (Pseudocode)
Input: Detection sets DL and DR, camera parameters (b, f) Output: Stereo object pairs P and their depths Z 1: procedure STEREO_MATCHING(DL, DR, b, f) 2:   // Initialize cost matrix 3:   C ← zeros(|DL|, |DR|) 4:   Z_temp ← zeros(|DL|, |DR|) 5: 6:   // Compute matching costs for all pairs 7:   for i = 1 to |DL| do 8:    for j = 1 to |DR| do 9:     // Assume correspondence and estimate depth 10:      Z_temp[i,j] ← TRIANGULATE(DL[i].center, DR[j].center, b, f) 11: 12:      // Calculate expected disparity 13:      d ← b × f/Z_temp[i,j] 14: 15:      // Warp bounding box and compute IoU 16:      bbox_warped ← WARP_BBOX(DL[i].bbox, d) 17:      C[i,j] ← −IoU(bbox_warped, DR[j].bbox) 18:     end for 19:    end for 20: 21:    // Find optimal assignment using Hungarian algorithm 22:    P ← HUNGARIAN_ASSIGNMENT(C) 23: 24:    // Filter matches by threshold and assign depths 25:    for each match (i,j) in P do 26:     if C[i,j] > −τ then //Since C contains negative IoU values 27:      Remove (i,j) from P 28:     else 29:      Z[i,j] ← Z_temp[i,j] 30:     end if 31:    end for 32: 33:    return P, Z 34: end procedure

### 3.4. Stage 3: Stereo Tracking with Temporal Data Association

The third stage performs stereo tracking with temporal data association to maintain consistent object IDs across frames while leveraging stereo correspondence information. As input, this stage receives the detected bounding boxes (DL and DR) and stereo object pairs (P) from the previous stages.

**Figure 3:**
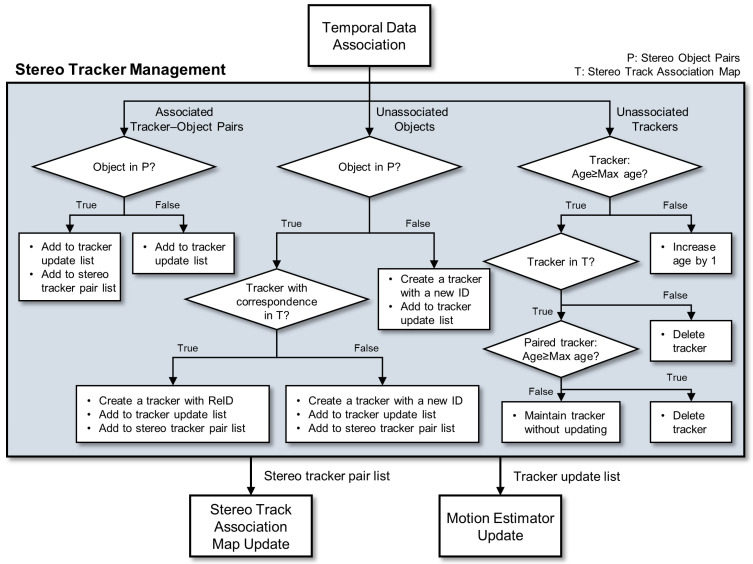
Flowchart of stereo tracker management in temporal data association. The process handles three scenarios based on association results: (1) Associated Tracker–Object Pairs, where trackers are updated with actual detection positions and added to both tracker update and stereo tracker pair lists; (2) Unassociated Objects, where new trackers are created with either re-identified IDs (if corresponding tracker exists in T) or new IDs, then added to appropriate lists; and (3) Unassociated Trackers, where age-based decisions determine whether to increment age, maintain without updating, or delete trackers based on their presence in the stereo track association map (T) and paired tracker status. P denotes stereo object pairs from the matching stage, and T represents the stereo track association map. The outputs include an updated stereo tracker pair list and tracker update list, which feed into the stereo track association map update and motion estimator update modules.

The tracking process begins with predicting the positions of tracked objects in the current frame using motion estimators that incorporate historical trajectory information [45]. Temporal data association then establishes correspondences between predicted tracker positions and detected objects using cost functions that incorporate IoU-based matching [46], the Hungarian algorithm [47], Kalman filter predictions [48], and feature-based similarity measures [36], among others. Notably, in the proposed framework, motion estimation and temporal data association are performed independently for each camera; that is, separate tracker instances operate on the left and right image streams, respectively. These trackers are then linked through the stereo track association map (denoted as T in Figure 3 and Algorithm 3), which maintains cross-view correspondences for the same physical object.

Figure 3 presents a detailed flowchart of the tracker management process, which follows three primary scenarios based on association results:

- Unassociated Trackers: When existing trackers fail to associate with any detected object, the system increments their age counter if the age is below the maximum threshold. As shown in the right branch of Figure 3, for trackers whose age equals or exceeds the maximum threshold, the system checks their status in the stereo track association map (denoted as T in Figure 3 and Algorithm 3). If the tracker exists in the map and its paired tracker also has an age equal to or exceeding the maximum threshold, both trackers are deleted. If the paired tracker’s age is below the threshold, the current tracker is maintained without updates to preserve stereo consistency. Trackers not included in the map are immediately deleted when their age reaches the maximum threshold [23].
- Unassociated Objects: The middle branch of Figure 3 illustrates the handling of detected objects that lack tracker associations. The system first verifies their stereo pairing status from P (denoted in Algorithm 2 and Figure 3). Unpaired objects generate new independent trackers. For stereo object pairs, if their corresponding object has an existing tracker in the stereo track association map, a new tracker is created for the object but inherits the ID from the previously tracked object, implementing re-identification that maintains consistent ID after temporary occlusions or truncations while starting fresh motion estimation from the current position [49]. Otherwise, a new tracker for the object is initialized with a new ID.
- Associated Tracker–Object Pairs: The left branch of Figure 3 shows that successfully associated pairs replace the predicted tracker position with the actual detected object position in the current frame. Specifically, while the motion estimator provides predicted positions based on historical trajectory information, the association confirms the actual object location, allowing the tracker state to be updated with this ground-truth position rather than relying on the prediction [1].

After the tracker management phase, as indicated in the bottom section of Figure 3, the system performs two distinct update operations. First, motion estimators are updated for all trackers that have associated objects, incorporating the current frame’s detection positions into their trajectory models for improved future predictions. Second, the stereo track association map is updated based on the current stereo object pairs from P, establishing new tracker correspondences or modifying existing ones to maintain consistency between left and right view trackers that follow the same physical object.

The modular design in tracker management allows integration with existing tracking algorithms for temporal data association and motion estimation components, providing flexibility in implementation while maintaining the benefits of stereo-aware tracking [50]. The complete stereo tracking process is described in Algorithm 3 using pseudocode notation.
**Algorithm 3:** Stereo Tracking (Pseudocode)
Input: Detection sets DL and DR, Stereo object pairs P, Tracker sets TL and TR, Association Map T (list) Output: Updated trackers and association map 1: procedure STEREO_TRACKING(DL, DR, P, TL, TR, T) 2:   // Predict tracker positions for all trackers 3:   for each tracker t in TL ∪ TR do 4:    t.predicted ← PREDICT(t.estimator) 5:   end for 6: 7:   // Temporal data association (returns tracker-object pairs) 8:   AL ← ASSOCIATE(TL, DL) //Associated tracker-object pairs for left 9:   AR ← ASSOCIATE(TR, DR) //Associated tracker-object pairs for right 10: 11:    // Process unassociated trackers 12:    for each tracker t in TL ∪ TR do 13:     if t not in TRACKERS(AL ∪ AR) then //t is not in associated trackers 14:      if t.age ≥ max_age then 15:       if EXISTS_IN_MAP(t, T) then 16:        paired_t ← GET_PAIRED_TRACKER(t, T) 17:        if paired_t.age ≥ max_age then 18:         DELETE(t) 19:         DELETE(paired_t) 20:        end if 21:       //Otherwise, maintain tracker without update 22:       else 23:        DELETE(t) 24:       end if 25:      else 26:       t.age ← t.age + 1 27:      end if 28:     end if 29:    end for 30: 31:    // Handle unassociated objects 32:    for each object o not in OBJECTS(AL ∪ AR) do //o is not in associated objects 33:     if HAS_STEREO_PAIR(o, P) then 34:      if PAIRED_TRACKER_EXISTS(o, P, T) then 35:       paired_tracker ← GET_PAIRED_TRACKER(o, P, T) 36:       // Create new tracker with re-identified ID 37:       t_new ← CREATE_TRACKER(o) 38:       t_new.id ← paired_tracker.id //Re-identification 39:       t_new.age ← 0 40:       if o ∈ DL then ADD_TO_TL(t_new) else ADD_TO_TR(t_new) 41:      else 42:       t_new ← CREATE_TRACKER(o) 43:       t_new.id ← GENERATE_NEW_ID() 44:       t_new.age ← 0 45:       if o ∈ DL then ADD_TO_TL(t_new) else ADD_TO_TR(t_new) 46:      end if 47:     else 48:      t_new ← CREATE_TRACKER(o) 49:      t_new.id ← GENERATE_NEW_ID() 50:      t_new.age ← 0 51:      if o ∈ DL then ADD_TO_TL(t_new) else ADD_TO_TR(t_new) 52:     end if 53:    end for 54: 55:    // Update associated trackers with actual detected positions 56:    for each (tracker t, object o) in AL ∪ AR do 57:     // Replace predicted position with actual detection 58:     t.position ← o.position 59:     t.age ← 0 //Reset age for associated trackers 60:     UPDATE_TRACKER_STATE(t, o) 61:    end for 62: 63:    // Update motion estimators for associated and new trackers only 64:    for each tracker t in TL ∪ TR do 65:     if (t.age = 0) then //Associated or newly created trackers 66:      UPDATE_MOTION_ESTIMATOR(t) 67:     end if 68:    end for 69: 70:    // Update stereo track association map (list structure) 71:    T ← UPDATE_STEREO_ASSOCIATIONS(P, TL, TR, T) 72: 73:    return TL, TR, T 74: end procedure

## 4. Results

### 4.1. Experimental Setup

The experimental platform for validating the proposed 2.5D multi-object tracking framework consists of a stereo vision system, computational hardware, and software environment configured for real-time processing.

#### 4.1.1. Stereo Vision System Configuration

**Figure 4:**
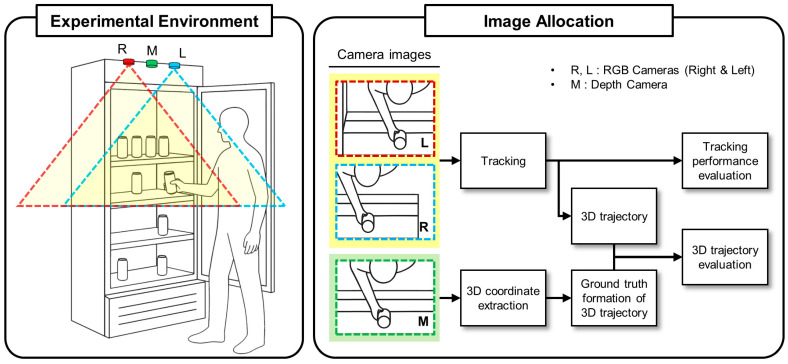
Experimental setup and data processing pipeline for 2.5D multi-object tracking validation. The left panel shows the physical configuration with stereo RGB cameras (L: left, R: right) and a depth camera (M: middle) mounted at the top of a commercial refrigerator. The yellow triangular regions outlined by red and cyan dashed lines indicate the fields of view for the left and right cameras, respectively. The right panel illustrates the image allocation and processing workflow: stereo RGB camera images are processed through the tracking system to generate 3D trajectories, which are then compared with ground truth from the depth camera for both tracking performance evaluation and 3D trajectory validation.

The stereo vision system was constructed using two identical RGB cameras, each equipped with a HU205 image sensor (Huentek Co., Ltd., Gwangju, Republic of Korea) and HULE51300 lens (Huentek Co., Ltd.). As illustrated in Figure 4, the stereo cameras (marked as L for left and R for right) were mounted at the top of a commercial refrigerator, oriented downward to capture the interior workspace. The cameras were mounted on a rigid baseline with parallel optical axes to ensure consistent stereo geometry. The stereo camera system was calibrated using the standard checkerboard calibration method [20] to obtain intrinsic parameters (focal length, principal point, distortion coefficients) and extrinsic parameters (rotation and translation between cameras). The baseline distance was set to 280 mm to provide an optimal balance between depth resolution and field of view overlap for indoor environments. The yellow triangular regions outlined by red and cyan dashed lines in Figure 4 represent the individual fields of view of the left and right cameras, respectively, with their overlapping area defining the effective stereo vision workspace.

Additionally, an Intel RealSense depth camera D435f (Intel Corp., Santa Clara, CA, USA), marked as M in Figure 4, was installed between the stereo cameras for validation purposes, as detailed in Section 4.4. This configuration enables direct comparison between the proposed stereo-based depth estimation and commercial depth sensing technology within the same workspace.

#### 4.1.2. Computational Platform

All experiments were conducted on a workstation with an Intel Core i9-12900K CPU, 64 GB of RAM, and an NVIDIA GeForce RTX 3090 GPU. The framework was implemented using Python 3.8, CUDA 11.8, and PyTorch 2.0. The system was designed to process stereo video streams at 30 frames per second (FPS), with the GPU handling object detection inference while the CPU managed stereo matching, temporal association, and tracker management tasks in parallel. This heterogeneous computing approach maximized throughput and minimized latency in the tracking pipeline.

### 4.2. Dataset Preparation

#### 4.2.1. Custom Dataset Construction

To comprehensively validate the proposed 2.5D stereo tracking framework, a custom stereo dataset tailored to commercial refrigerator environments was constructed. The custom dataset considered three primary aspects: First, public datasets are collected under varying camera setups and calibration conditions, which exhibit geometric inconsistencies across sequences. Such variability makes it difficult to isolate algorithmic performance from dataset-induced bias. In contrast, our custom dataset ensures consistent geometric properties by employing identical stereo configuration and unified calibration and rectification across all sequences.

Second, quantitative evaluation of 3D trajectory accuracy requires depth ground truth that is spatially and temporally aligned with stereo image pairs. However, existing public datasets either provide only 2D annotations or rely on LiDAR depth that is not synchronized with stereo inputs. To address this limitation, our dataset includes ground-truth depth obtained by synchronizing a stereo camera system with a calibrated depth sensor, enabling accurate 3D validation.

Third, the dataset was designed to reflect realistic interaction scenarios with frequent occlusions and truncations, which commonly occur in hand–object interaction tasks but are underrepresented in public benchmarks designed primarily for autonomous driving (e.g., KITTI). By incorporating near-field interactions and dense object clutter, the dataset enables rigorous evaluation of the robustness of the stereo-based identity preservation mechanism.

The dataset comprises three subsets for different validation purposes: (1) dataset for object detection model training and validation (Section 4.2.2), (2) dataset for 3D position accuracy evaluation (Section 4.4), and (3) dataset for tracking performance evaluation (Section 4.5).

#### 4.2.2. Data Collection and Annotation

Data were collected using both cameras of the stereo vision system, and hand instances in each view were annotated with bounding boxes. The annotation process was conducted using Roboflow [51], a web-based annotation platform that provides efficient tools for bounding box labeling and dataset management. The collected dataset comprises 26,226 images with 52,934 annotated hand instances, divided into training (20,991 images, 80%), validation (4207 images, 16%), and test (1028 images, 4%) sets.

All images were captured at 640 × 480 resolution. The dataset captures various hand gestures, including reaching, grasping, and retrieving products at different shelf locations within the 300–2000 mm working range, exhibiting significant scale variations. The dataset includes challenging detection scenarios such as partial occlusions caused by refrigerator products and shelves during hand–product interactions, as well as truncations at image boundaries when hands enter or exit the field of view.

### 4.3. Implementation Details

#### 4.3.1. Object Detection Model Selection and Training

For the object detection component of the 2.5D multi-object tracking framework, YOLOv11-Large [19] was selected as the deep learning-based detection model. While YOLOv12 with attention mechanisms represents the latest advancement in the YOLO series, YOLOv11 was chosen based on its faster inference speed. Comparative benchmarks validate this selection [52]: YOLOv11-Large achieves comparable accuracy (mAP@0.5:0.95 53.3%) to YOLOv12-Large (mAP@0.5:0.95 53.7%, +0.4%) while providing approximately 9% faster inference speed (6.2 ms vs. 6.77 ms), confirming that YOLOv11-Large offers an optimal balance.

The training process achieved exceptional detection performance, with the validation set yielding a mean Average Precision of 0.993 at IoU threshold 0.5 (mAP@0.5) and 0.794 for the stricter mAP@0.5:0.95 metric. These results were consistently reproduced on the test set, achieving mAP@0.5 of 0.993 and mAP@0.5:0.95 of 0.789, demonstrating robust generalization without overfitting. The high mAP@0.5 indicates excellent detection reliability, which is crucial for tracking initialization, while the mAP@0.5:0.95 score confirms precise bounding box localization, which is necessary for accurate stereo matching.

#### 4.3.2. Tracking Algorithm Implementation

Temporal data association and motion estimation components were implemented, adjusting OC-SORT [37] through its modules of Observation-Centric Momentum (OCM) and Observation-Centric Reassociation (OCR). The algorithm significantly reduces association errors through its OCM module, which maintains object momentum during nonlinear motion by computing velocity consistency from historical trajectories. This observation-centric approach provides better noise robustness compared to traditional state-space models, as it directly leverages detection observations rather than relying solely on motion predictions. In addition, OCR effectively reduces ID switches during temporary occlusion situation by re-associating with the previous observation information in the detection-to-track association. Furthermore, OC-SORT’s modular design enables seamless integration with various detection backends without requiring extensive parameter tuning, making it ideal for the proposed stereo tracking framework.

In our implementation, additional observation consistency terms were incorporated into the association cost function of OCM to enhance tracking stability: center distance, orientation consistency, and velocity discrepancy penalty. These terms penalize associations that violate motion continuity constraints, suppressing physically implausible 2D movements and improving inter-frame tracking stability.

The data association process implementation employed a two-stage matching strategy. The association threshold was set to 0.3 IoU for high-confidence matches, with a secondary threshold of 0.15 for recovery association. Furthermore, to exploit stereo geometry constraints, the disparity difference between consecutive frames was limited to not exceed 40 pixels, eliminating geometrically inconsistent matches that violate depth continuity.

#### 4.3.3. 2.5D Multi-Object Tracking Framework Integration

The complete framework was implemented as a modular Python package designed for real-time stereo vision processing. Prior to the main processing pipeline, comprehensive image preprocessing was performed to compensate for hardware imperfections inherent in the stereo vision system. The preprocessing step involved camera calibration, lens distortion correction, and stereo rectification for the image pairs. Following preprocessing, the stereo processing pipeline began with hardware-triggered camera synchronization, achieving a sub-ms temporal offset between left and right captures. Both rectified images were then processed through the YOLOv11 detector in parallel using batch inference to maximize GPU utilization.

The stereo matching implementation employed DLT-based triangulation, computing 3D positions for all possible object pair combinations between the two views. Disparity-based validation with adaptive thresholds ensured geometric consistency; the expected disparity derived from triangulated depth was compared against actual bounding box positions. The Hungarian algorithm determined optimal one-to-one correspondences with an IoU threshold of 0.01 for accepting valid stereo matches. This deliberately low threshold was selected to ensure that small objects, particularly hands at far distances or partially visible at frame edges, were not missed during the stereo matching process, as even minimal overlap can indicate valid correspondence given accurate triangulation.

The tracker management system maintained dual tracker pools for left and right views, with a stereo track association map implemented as a bidirectional dictionary structure enabling efficient lookup of corresponding trackers. Trackers were allowed a maximum age of 30 frames (equivalent to 1 s at 30 FPS) before deletion, providing sufficient time for re-identification after temporary occlusions. A re-identification buffer preserved tracker information indefinitely until successful re-identification occurred, enabling the system to restore consistent IDs when objects reappeared even after extended disappearances.

Real-time performance optimization was achieved through an optimized architecture where detection, matching, and tracking operations were executed in a single process. The GPU-CPU pipeline implemented asynchronous data transfers to minimize latency, while frame buffering accommodated occasional processing delays without dropping frames. This optimized implementation achieved processing rates up to 70 FPS on the specified hardware configuration, providing substantial headroom for additional processing tasks or handling of more complex scenes. The framework exposed a streamlined API for integration with downstream applications, returning tracked objects with 2.5D positions, velocities, and unique IDs maintained consistently throughout the tracking session.

### 4.4. Depth Estimation Accuracy Evaluation

#### 4.4.1. Validation Methodology

To evaluate the 3D position estimation accuracy of the proposed 2.5D multi-object tracking framework, a concomitant validation method was employed using a commercial depth camera as ground-truth reference. The depth camera introduced in Section 4.1 was selected for validation due to its high depth accuracy (<2% at 2000 mm) and active stereo technology with an infrared projector that provides reliable depth measurements independent of ambient lighting conditions. It features a depth resolution of 1280 × 720 at 30 FPS with a depth range of 300 to 3000 mm, well-suited for the refrigerator monitoring application. Using a separate depth camera ensured that the evaluation of stereo triangulation accuracy was independent of the stereo vision system itself, avoiding circular validation.

The validation setup involved a depth camera adjacent to the stereo vision system with overlapping fields of view covering most of the monitored volume, as illustrated in Figure 4. To obtain an independent depth reference for evaluating stereo triangulation accuracy, we generated depth-assisted ground truth using the RGB-D measurements from the depth camera. Both systems were temporally synchronized to acquire data simultaneously. The depth camera provided RGB images with spatially aligned dense depth maps, while the stereo vision system estimated 3D object positions through the proposed stereo matching and DLT-based triangulation procedures.

During ground-truth generation, hand regions were manually annotated in the RGB images of the depth camera to ensure independence from the stereo-based detection results. For each annotation, a small region of interest (ROI) was defined around the box center and mapped onto the corresponding aligned depth map. Valid pixels within the ROI of the aligned depth map were back-projected into 3D space using the intrinsic parameters, and their mean coordinates were used as the reference position. ROI averaging reduces sensor noise and mitigates missing-depth artifacts near object boundaries, resulting in more stable ground-truth estimation. These depth-camera-based reference values are regarded as pseudo ground truth for validating the stereo-derived 3D positions, as both systems operate synchronously but differ in sensing modalities.

#### 4.4.2. Experimental Results

The validation experiment was conducted using four distinct video sequences capturing various hand movement patterns within the refrigerator environment with successfully tracked objects. Each sequence was approximately 20 s in duration, captured at 30 FPS. To ensure reliable one-to-one matching between depth camera and stereo camera observations, each sequence captured a single hand performing various motions across the entire working range (300–2000 mm depth), including reaching, grasping, and retrieving movements at different distances and positions. The 3D positions of tracked hand objects in each sequence were quantitatively compared with the depth camera-based ground-truth coordinates generated using the method described in Section 4.4.1.

**Figure 5:**
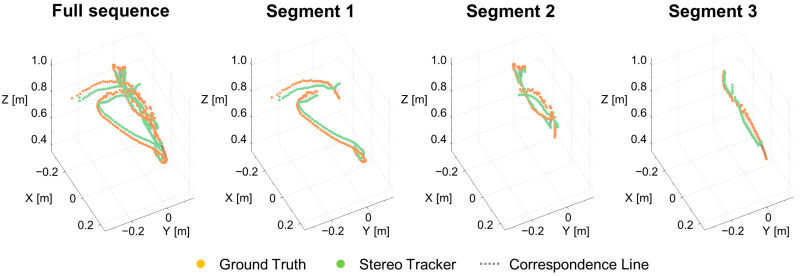
Comparison of 3D hand trajectories between ground truth (depth camera, orange dots) and the proposed stereo tracker (green dots) with correspondence lines (dotted gray) connecting matched points. The figure shows the full sequence and three detailed segments, demonstrating close alignment between the two systems across different motion patterns. The axes represent the 3D workspace in meters, with the origin at the stereo camera position.

Figure 5 presents a representative trajectory comparison from one validation sequence, demonstrating the close alignment between ground-truth (depth camera) and stereo tracker estimates across the full sequence and three detailed segments. The visualization reveals that the stereo tracking system accurately captures the overall motion pattern and maintains consistent depth estimation throughout the trajectory, with minor deviations primarily occurring during rapid movements or at the workspace boundaries.

Quantitative analysis across all four sequences yielded the following error metrics for 3D Euclidean distance ($d_{i}=\;\left|\left|p_{i}^{est}-\;p_{i}^{gt}\right|\right|$, where $p_{i}^{est}$ and $p_{i}^{gt}$ are the estimated and ground-truth 3D positions) between estimated and ground-truth positions: Root Mean Square Error ($\sqrt{\frac{1}{N}\sum_{i=1}^{N}{d_{i}}^{2}}$), Mean Absolute Error ($\frac{1}{N}\sum_{i=1}^{N}d_{i}$), median error ($median\;of\;\left\{d_{1},\cdots ,d_{n}\right\}$), 95th percentile error ($95th\;percentile\;of\;\left\{d_{1},\cdots ,d_{n}\right\}$). The Root Mean Square Error (RMSE) of 74.2 mm indicates the overall accuracy, including outliers, while the Mean Absolute Error (MAE) of 60.5 mm provides a more robust measure less sensitive to occasional large errors. The median error of 50.1 mm demonstrates that half of all measurements achieve accuracy better than 50 mm, suitable for hand tracking in retail applications. The 95th percentile error (P95) of 136.4 mm indicates that 95% of estimates fall within 136.4 mm of ground truth, with larger errors typically occurring at maximum reach distances where stereo baseline limitations become more pronounced.

These results validate that the proposed stereo matching module achieves depth estimation accuracy comparable to commercial depth sensors while operating solely on passive RGB imagery, eliminating the need for active infrared projection that may interfere with other sensors or violate privacy regulations in retail environments. The consistent sub-100 mm accuracy for the majority of measurements confirms the framework’s suitability for practical deployment in commercial refrigerator monitoring applications.

### 4.5. Multi-Object Tracking Performance

#### 4.5.1. Evaluation Dataset and Metrics

While Section 4.4 validated the 3D coordinate accuracy of the stereo matching module, a comprehensive evaluation of the stereo tracker’s robustness to occlusions and truncations is essential for practical deployment. To this end, a challenging test dataset was created consisting of 10 video sequences, each 20–25 s in duration, specifically designed to include frequent hand occlusions and truncations typical in refrigerator interaction scenarios. The sequences were captured at 640 × 480 resolution at 30 FPS using the stereo camera system with a 280 mm baseline. All hand objects in these sequences were manually annotated to establish ground truth for tracking evaluation.

The tracking performance was evaluated using standard multi-object tracking metrics that comprehensively assess different aspects of tracking quality. MOTA (Multiple Object Tracking Accuracy) combines false positives, false negatives, and ID switches into a single accuracy measure [35]. IDF1 (Identification F1-Score) evaluates the tracker’s ability to maintain correct IDs over time [53]. HOTA (Higher Order Tracking Accuracy) balances detection and association performance at multiple IoU thresholds [6]. DetA (Detection Accuracy) and LocA (Localization Accuracy) measure the spatial accuracy of detections, while AssA (Association Accuracy) and AssR (Association Recall) evaluate the temporal consistency of ID assignments [6].

#### 4.5.2. Comparative Analysis

Table 1 presents the tracking performance comparison between the proposed StereoSORT and five state-of-the-art monocular tracking algorithms: SORT, DeepSORT, ByteTrack, BoT-SORT (Bag of Tricks for SORT), and OC-SORT. The proposed StereoSORT achieves superior performance across all evaluation metrics, demonstrating the effectiveness of leveraging stereo information for robust tracking.

StereoSORT attains the highest MOTA score of 0.932, representing a 0.6–6.0 percentage point improvement over monocular methods, with the most significant gains over ByteTrack (0.874). The IDF1 score of 0.823 substantially exceeds all baselines, with improvements ranging from 5.8 percentage points over OC-SORT to 22.2 percentage points over DeepSORT, indicating superior ID preservation during occlusions. The HOTA score of 0.844 confirms balanced performance in both detection and association tasks.

The association metrics reveal the key advantage of stereo tracking, with StereoSORT achieving AssA of 0.775 and AssR of 0.787, significantly outperforming the best monocular method (OC-SORT) by 7.2 and 7.6 percentage points, respectively. This improvement directly results from the stereo tracker’s ability to maintain object ID when one view experiences occlusion while the other maintains visibility. The near-perfect LocA score of 0.981 demonstrates that stereo triangulation provides more accurate spatial localization compared to monocular depth estimation.

These results validate that the proposed stereo tracking module effectively leverages redundant visual information to achieve robust performance in challenging scenarios with frequent occlusions and truncations, making it particularly suitable for commercial refrigerator monitoring applications where hands frequently disappear behind products or shelves.

**Figure 6:**
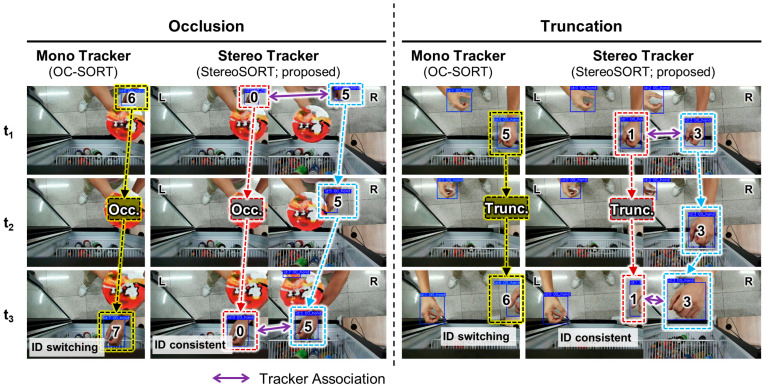
Qualitative comparison of tracking performance between the monocular tracker (OC-SORT) and the proposed stereo tracker (StereoSORT) under challenging scenarios. The left panel demonstrates occlusion handling where a hand temporarily disappears (Occ.) behind products, showing ID switching in the mono tracker (ID 6 → ID 7) while the stereo tracker maintains consistent IDs (0 and 5). The right panel illustrates truncation cases (Trunc.) at image boundaries, where the mono tracker exhibits ID inconsistency (ID 5 → ID 6) while the stereo tracker preserves stable IDs (1 and 3) through stereo correspondence. Purple arrows indicate tracker associations between views. The yellow, red, and blue bounding boxes represent different tracked objects with their respective IDs.

Beyond quantitative metrics, qualitative analysis provides crucial insights into the practical advantages of stereo tracking in handling challenging scenarios. Figure 6 illustrates representative examples comparing the proposed StereoSORT with OC-SORT (the best-performing monocular baseline) under occlusion and truncation conditions.

To quantify the specific contribution of leveraging stereo redundancy, the proposed system was evaluated in both monocular and stereo configurations. The monocular configuration utilizes only the left camera, thus excluding the Stereo Matching and Stereo Tracker Management modules. The stereo configuration demonstrated improved performance across all metrics. Notably, IDF1 increased from 0.772 to 0.823 (5.1 percentage points), with corresponding improvements in AssA (5.8 percentage points) and AssR (5.8 percentage points). In contrast, MOTA (0.932 vs. 0.931) and LocA (0.981 vs. 0.981) remained nearly identical, indicating that stereo matching and tracker management enhance ID consistency by reducing ID switches and fragmentation without degrading detection performance. Additionally, StereoSORT in monocular configuration showed improved performance over baseline OC-SORT [37] in key metrics. Specifically, AssA improved from 0.703 to 0.720 (1.7 percentage points) and HOTA from 0.802 to 0.814 (1.2 percentage points), demonstrating that the observation-consistency term (Section 4.3.2) contributes to tracking robustness.

In the occlusion scenario (left panel), a hand temporarily disappears behind products between frames t_1_ and t_3_. The monocular tracker exhibits ID switching, assigning new IDs when the hand reappears (ID 6 → ID 7), disrupting trajectory continuity. In contrast, StereoSORT maintains consistent IDs throughout the sequence by leveraging visibility in the alternate view when one camera’s view is occluded. The tracker association arrows demonstrate how stereo correspondence enables ID preservation even during complete occlusion in one view.

The truncation scenario (right panel) presents hands partially visible at image boundaries, a common occurrence when customers reach into the refrigerator from different angles. The monocular tracker struggles with ID consistency (0 and 5), showing frequent switches, particularly for the partially visible hand at the frame edge. StereoSORT successfully maintains stable IDs (1 and 3) by utilizing the complementary view, where the truncated object may be more completely visible, or by maintaining stereo correspondence even with partial observations.

These visual examples confirm that the stereo tracking module’s re-identification mechanism effectively exploits redundant visual information to maintain ID consistency. The ability to preserve tracker IDs during temporary occlusions and truncations is critical for applications requiring accurate trajectory analysis, such as customer behavior monitoring or interaction pattern recognition in retail environments. This qualitative evidence, combined with the superior quantitative metrics, demonstrates that StereoSORT provides robust and reliable tracking performance suitable for deployment in challenging real-world scenarios.

## 5. Discussion

The experimental results demonstrate that the proposed 2.5D stereo multi-object tracking framework achieves robust performance in challenging real-world scenarios with frequent occlusions and truncations. The superior tracking metrics, particularly the IDF1 score of 0.823 compared to 0.765 for the best monocular baseline (OC-SORT), validate our hypothesis that stereo vision’s redundant viewpoints can effectively mitigate single-view detection failures.

### 5.1. Analysis of Stereo Matching Performance

The depth estimation accuracy evaluation reveals that our lightweight DLT-based stereo matching approach achieves a median error of 50.1 mm, comparable to commercial depth sensors while operating solely on passive RGB imagery. This performance is particularly noteworthy considering the computational efficiency gained by avoiding dense stereo matching algorithms. The slightly higher RMSE of 74.2 mm indicates occasional outliers, primarily occurring at maximum reach distances where the stereo baseline becomes a limiting factor. For a refrigerator monitoring application with typical interaction distances of 300–1500 mm, this accuracy level is more than sufficient for reliable hand tracking.

The deliberate choice of a low IoU threshold (0.01) for stereo matching acceptance deserves further discussion. While counterintuitive, this threshold ensures that small or partially visible objects at frame boundaries maintain stereo correspondence. Our experiments showed that higher thresholds (0.1–0.3) resulted in frequent loss of stereo pairs for truncated objects, defeating the purpose of leveraging stereo redundancy. However, we acknowledge that using bounding box centers for DLT-based triangulation assumes these points correspond to the same 3D location, which may not hold perfectly for non-rigid objects like hands or for large objects at close distances. This assumption has minimal impact during the stereo object matching stage, as matching is based on the entire bounding box region rather than a single point. However, when computing actual 3D positions through triangulation, depth errors can occur if the two center points do not represent the same physical location. In our application domain (hands at 300–2000 mm distances), this assumption provides sufficient accuracy as evidenced by the median depth error of 50.1 mm (Section 4.4.2). For applications involving larger non-rigid objects or closer distances, incorporating multiple correspondence points (e.g., corner points or skeleton-based keypoints) could improve accuracy.

### 5.2. Robustness to Occlusions and Truncations

The significant improvement in association metrics (AssA: 0.775 vs. 0.703 for OC-SORT) directly demonstrates the effectiveness of our dual-tracker approach. When one view experiences occlusion, the corresponding tracker in the alternate view maintains object ID, enabling seamless re-identification when visibility is restored. This mechanism proves particularly valuable in retail environments where products and shelving create frequent occlusions.

The qualitative analysis in Figure 6 reveals an interesting pattern: monocular trackers tend to exhibit ID switches not only during occlusions but also immediately after recovery. This suggests that appearance-based re-identification alone is insufficient when objects undergo partial visibility changes. In contrast, our stereo correspondence provides a geometric constraint that maintains ID consistency independent of appearance variations.

### 5.3. Computational Efficiency Considerations

The framework achieves a processing rate of 70 FPS, demonstrating the practical advantage of our 2.5D representation over full 3D MOT systems. By avoiding computationally expensive 3D bounding box regression and dense stereo matching, the system maintains real-time performance while providing essential depth information. The per-frame latency is 13.6 ms in total, comprising detector inference (12.3 ms on GPU) and tracking (1.25 ms on CPU, including 0.23 ms for stereo matching). Notably, excluding detection, the tracking module runs entirely on the CPU within 1.25 ms without any appearance feature extraction or deep Re-ID computation, demonstrating the lightweight design of the proposed method. The GPU was used only to accelerate object detection during evaluation and is not required by the tracking pipeline itself. For future optimization, lightweight backbones may be explored to enable fully CPU-based embedded deployment.

### 5.4. Limitations and Practical Considerations

Despite the strong performance, several limitations warrant discussion.

First, the stereo baseline of 280 mm, while suitable for indoor environments, may be insufficient for larger spaces requiring an extended depth range. The system’s depth estimation accuracy degrades beyond 2000 mm due to the fixed baseline limitation.

Second, the system’s performance degrades in scenarios with significant lighting variations between stereo views, as this violates the photometric consistency assumption underlying stereo matching. In our experiments, we observed matching failures when one camera faced direct illumination while the other was shadowed, resulting in incorrect correspondences and depth estimation errors exceeding 200 mm.

Third, the method struggles when objects are simultaneously occluded or truncated in both stereo views, as the redundancy advantage is lost. In such cases, the system reverts to monocular tracking behavior until visibility is restored in at least one view.

Fourth, rapid hand movements can cause motion blur in the captured images, leading to inaccurate bounding box detections and subsequent stereo matching failures. The DLT-based triangulation relies on precise center point localization, which degrades under motion blur conditions.

Fifth, due to parallax or partial occlusion, the same object can be perceived differently across the left and right views. For example, two objects may be distinctly separated and detected in one view but merged into a single bounding box in the other. If this merged box is incorrectly matched to the wrong object in the opposite view, erroneous correspondences can produce a mismatched tracker pair. When the objects later separate, ID reuse may fail, resulting in track fragmentation. Because the current system updates the stereo tracker association map on a per-frame basis, it is not sufficiently robust for such transient merging and splitting events. Future work could incorporate temporal association history or continuity constraints to maintain a more stable correspondence between stereo trackers.

## 6. Conclusions

This paper presented a real-time robust 2.5D stereo multi-object tracking system that effectively addresses the challenges of occlusions and truncations. The key contribution lies in demonstrating that lightweight stereo matching based solely on bounding box coordinates, combined with intelligent tracker management, can achieve superior tracking performance compared to sophisticated monocular methods.

The proposed framework makes three significant contributions to the field. First, the resource-aware stereo matching algorithm eliminates the computational overhead of appearance feature extraction while maintaining robust correspondence through geometric constraints. Second, the resilient dual-tracker design with re-identification capability ensures tracking continuity even under frequent detection failures. Third, the 2.5D representation provides a practical balance between computational efficiency and spatial awareness, making the system suitable for real-time deployment.

Experimental validation on a custom refrigerator monitoring dataset demonstrated that StereoSORT achieves MOTA of 0.932 and IDF1 of 0.823, substantially outperforming state-of-the-art monocular trackers. The depth estimation accuracy (median error: 50.1 mm) proves sufficient for practical applications while maintaining real-time performance at 70 FPS.

Several important directions for future research emerge from this work. First, comprehensive benchmarking on public datasets such as KITTI is essential to validate the framework’s generalizability beyond the current application domain. Although this study focused on a single-class setting, the proposed method does not limit the applicability because it is geometry-based and does not rely on class-dependent appearance cues. Future work will evaluate the framework on large-scale stereo benchmarks to further assess its scalability across diverse stereo configurations, object types, and operating environments.

Second, extending the framework to multi-class scenarios introduces additional challenges beyond dataset-level generalization. In particular, stereo matching must remain reliable even when detections from the two views belong to different semantic classes due to detector uncertainty or classification noise. To address such ambiguity, two promising strategies can be explored: (1) adopting the class with the highest confidence score from either view, or (2) selecting the class with the highest mean confidence across both views for balanced fusion. This extension is critical for deploying the framework in complex environments involving complex object categories. Moreover, incorporating explainable AI techniques could enhance decision reliability by providing interpretable evidence for class assignment (e.g., saliency and Grad-CAM heatmaps), which would improve transparency in real-world applications [55,56,57,58].

Third, the current motion estimation operates in the 2D image space, which may lead to physically inconsistent predictions for objects moving along the depth axis. This design choice was made to prioritize real-time efficiency and maintain temporal stability during the mechanism-level validation stage of this study. However, a more principled extension would incorporate a 3D motion model that performs prediction in world coordinates while leveraging stereo-based depth information, followed by projection back to the image plane for association. Such a depth-aware motion model would provide physically consistent trajectory estimation and improve accuracy for objects exhibiting significant depth variation. In future work, the extension to 3D MOT can be achieved by integrating existing stereo-based 3D position estimates with a 3D Kalman filter or velocity-aware motion models.

Additionally, investigating adaptive stereo baseline configurations could optimize the depth-resolution trade-off for different operational ranges. The integration of temporal depth consistency constraints could further improve tracking robustness during extended occlusions. From a deployment perspective, developing hardware-accelerated implementations for edge devices would enable broader adoption in resource-constrained environments.

The framework’s principles demonstrate potential for adaptation to other domains requiring robust tracking, such as industrial automation, sports analytics, and assisted living environments. As stereo vision systems become increasingly prevalent in robotics and autonomous systems, the proposed lightweight yet robust tracking approach offers a practical solution for real-world deployment where computational resources and reliability are equally critical.

## Figures and Tables

**Table 1:** Quantitative comparison of multi-object tracking performance between the proposed StereoSORT and state-of-the-art monocular trackers on the occlusion/truncation test dataset. All metrics range from 0 to 1, with higher values (↑) indicating better performance. The proposed method achieves superior performance across all evaluation metrics, demonstrating the effectiveness of stereo-based tracking.

Tracker	MOTA (↑)	IDF1 (↑)	HOTA (↑)	DetA (↑)	AssA (↑)	AssR (↑)	LocA (↑)
SORT [45]	0.920	0.651	0.665	0.828	0.535	0.550	0.909
DeepSORT [36]	0.920	0.601	0.692	0.912	0.525	0.529	0.981
ByteTrack [24]	0.874	0.609	0.616	0.775	0.492	0.510	0.888
BoT-SORT [54]	0.929	0.624	0.694	0.892	0.540	0.548	0.949
OC-SORT [37]	0.926	0.765	0.802	0.915	0.703	0.711	0.982
StereoSORT (proposed)	0.932	0.823	0.844	0.919	0.775	0.787	0.981

## Data Availability

The sample data and codes presented in the study are available in GitHub: https://github.com/vPrismLab/StereoTracker (accessed on 18 September 2025). The training datasets are available from the corresponding author upon request, for research purposes.

## Abbreviations

The following abbreviations are used in this manuscript:

AssA	Association Accuracy
AssR	Association Recall
BoT-SORT	Bag of Tricks for Simple Online and Realtime Tracking
CPU	Central Processing Unit
DeepSORT	Deep Simple Online and Realtime Tracking
DetA	Detection Accuracy
DLT	Direct Linear Transform
FPS	Frames per second
GPU	Graphics Processing Unit
HOTA	Higher Order Tracking Accuracy
ID	Identity
IDF1	Identification F1 Score
IoU	Intersection over Union
LocA	Localization Accuracy
MAE	Mean Absolute Error
MOT	Multi-Object Tracking
MOTA	Multiple Object Tracking Accuracy
OCM	Observation-Centric Momentum
OCR	Observation-Centric Reassociation
OC-SORT	Observation-Centric SORT
P95	95th Percentile Error
RMSE	Root Mean Square Error
SORT	Simple Online and Realtime Tracking
YOLO	You Only Look Once
